# Supplementary material for: Quality of Life in COVID-Related ARDS Patients One Year after Intensive Care Discharge (Odissea Study): A Multicenter Observational Study
Source: J Clin Med. 2023 Jan 29;12(3):1058. doi: 10.3390/jcm12031058 (PMC9918008; doi:10.3390/jcm12031058)
Supplement: Supplementary file 1 [file jcm-12-01058-s001.zip › jcm-2176037-supplementary.pdf]

Supplementary Material S1.

SF-36 and IES-R "pocket guide".

SF-36 parameter	Number of items	Meaning	Score range
**Physical Functioning (PF)**	10	Extent to which health interpheres with physical activity such as walking, climbing stairs, bathing, dressing, sports. The higher the score, the most vigorous activities could be done.	**0-100** The higher the score, the better the quality of life
**Role Physical (RP)**	4	Extent to which health interpheres with usual daily activities, such as work, housework, or school.	
**Role Emotional (RE)**	3	Extent to which health interpheres with usual daily social activities; for example, accomplished less than would like	
**Bodily Pain (BP)**	2	Extent of bodily pain in past four weeks	
**General Health (GH)**	5	Overall rating of current health in general	
**Vitality (VT)**	4	Energy or tiredness	
**Social Functioning (SF)**	2	Extent to which health interpheres with normal social activities, such as visiting friends.	
**Mental Health (MH)**	5	General mood of affect, including depression, anxiety and psychological well-being.	

**Health Change (HC)**	1	Change in health status compared to one year before.	
**Mental Component Summary (MCS)**	Weighted MH+RE+SF+VT+GH scores	Global mental health condition	
**Physical Component Summary (PCS)**	Weighted PF+PR+BP+VT+GH scores	Physical wellness	

MCS and PCS, which are two summary scores including all the SF-36 items, are constructed using principal component analysis, basing on the data of the general population of the US standardized to obtain a mean of 50 (considered the normal threshold) and a standard deviation of 10.

Reference: Ware JE, Kosinski M, Bayliss MS, McHorney C, Rogers WH, Raczek A. Comparison of methods for scoring and statistical analysis of the SF-36 health profile and summary measures: summary of results from the Medical Outcomes Study. Med Care. 1995;33:AS264–79.

IES-R	Number of items	Meaning	Score range
**Intrusion**	8	This item considers repeated thoughts about the trauma, intrusive thoughts, nightmares, intrusive feelings and imagery, dissociative-like re-experiencing.	**0-32**<br><br>The higher the score, the higher the probability to have PTSD
**Avoidance**	8	This item considers effortful avoidance of situations that serve as reminders of the trauma, symptoms involving reduced involvement with the external world, numbing of responsiveness, avoidance of feelings, situations, and ideas.	
**Hyperarousal**	6	This item considers anger, irritability, hypervigilance, difficulty	

		concentrating, heightened startle.	**0-24**<br>The higher the score, the higher the probability to have PTSD

Each item can assume a value from 0 to 4, for a total maximum score of 88.
Sum scores equal to or greater than 33 or mean cut-off value equal to or greater than 1.75 for overall questions indicate the probable presence of PTSD.
Reference: Weiss, D.S. (2007). The Impact of Event Scale: Revised. In J.P. Wilson & C.S. Tang (Eds.), Cross-cultural assessment of psychological trauma and PTSD (pp. 219-238). New York: Springer.

Supplementary Material S2.

Univariable and multivariable analysis to find possible correlations between SF-36 items and baseline, medical and ICU data.

	**Univariable Analysis**			**Multivariable Analysis**		
**PF**	**Beta**	**95% CI**	**p-value**	**Beta**	**95% CI**	**p-value**
**Age**	-0.41	-0.69,-0.13	**0.004**	-0.07	-0.39,0.26	0.681
**Female sex**	-7.87	-14.71,-1.03	**0.024**	-6.97	-13.74,-0.19	**0.044**
**High level of education**	2.86	-2.98,8.70	0.336			
**Married/Cohabiting**	-1.89	-8.64,4.86	0.582			
**Employment**						
Active worker vs jobless	21.69	10.94,32.43	**<0.001**	10.81	-0.78,22.41	0.068
Retired vs jobless	17.83	7.08,28.59	**0.001**	9.79	-1.55,21.12	0.090
Retired vs active worker	-3.85	-9.73,2.02	0.197	-1.03	-7.38,5.33	0.751
**Weight at baseline**	-0.03	-0.20,0.13	0.694			
**Weight at FUP**	-0.08	-0.28,0.12	0.438			
**Weight variation**	0.26	-0.24,0.77	0.305			
**APACHE II score**	-0.49	-1.25,0.27	0.206			

**ALI score**	0.38	-6.16,6.91	0.910			
**Cardiovascular disease**	-11.58	-17.12,-6.04	**<0.001**	-10.02	-15.71,-4.34	**0.001**
**Pulmonary disease**	-7.32	-14.97,0.33	0.061			
**Kidney disease**	-5.86	-18.71,6.98	0.370			
**Liver disease**	-19.88	-36.93,-2.84	**0.022**	-17.07	-33.05,-1.10	**0.036**
**Diabetes**	-9.99	-18.75,-1.24	**0.025**	-3.37	-12.0,5.26	0.443
**Length of hospital stay before ICU admission**	0.26	-0.46,0.98	0.478			
**LOS_{ICU}**	-0.11	-0.32,0.11	0.325			
**LOS_{HOSP}**	-0.22	-0.35,-0.08	**0.001**	-0.21	-0.34,-0.08	**0.001**
**Steroid administration**	-0.64	-7.88,6.60	0.863			
**NMB administration**	7.13	-1.32,15.58	0.098			
**RRT in ICU**	-8.84	-20.28,2.61	0.130			
**Tracheostomy in ICU**	-5.23	-11.99,1.53	0.129			
**MV duration**	-0.05	-0.34,0.23	0.704			

	**Univariable Analysis**			**Multivariable Analysis**		
**RP**	**Beta**	**95% CI**	**p-value**	**Beta**	**95% CI**	**p-value**
**Age**	-0.40	-0.86,0.07	0.094			
**Female sex**	-6.60	-18.27,4.35	0.227			
**High level of education**	5.82	-3.43,15.06	0.217			
**Married/Cohabiting**	-7.36	-17.97,3.26	0.174			
**Employment**						
Active worker vs jobless	16.34	-0.88,33.56	0.063	13.22	-4.94,31.39	0.153
Retired vs jobless	17.36	0.11,34.6	**0.049**	13.76	-4.35,31.87	0.136
Retired vs active worker	1.01	-8.45,10.48	0.833	0.54	-8.94,10.01	0.911
**Weight at baseline**	-0.05	-0.31,0.22	0.732			
**Weight at FUP**	-0.03	-0.34,0.28	0.857			
**Weight variation**	0.46	-0.30,1.23	0.235			

**APACHE II score**	-0.66	-1.91,0.59	0.302			
**ALI score**	-6.30	-16.86,4.27	0.242			
**Cardiovascular disease**	-3.52	-12.95,5.90	0.463			
**Pulmonary disease**	-8.23	-21.01,4.55	0.206			
**Kidney disease**	-2.45	-24.07,19.17	0.824			
**Liver disease**	-41.50	-68.60,-14.41	**0.003**	-40.16	-67.19,-13.13	**0.004**
**Diabetes**	-10.99	-25.63,3.65	0.141			
**Length of hospital stay before ICU admission**	-0.26	-1.47,0.95	0.673			
**LOS_{ICU}**	-0.28	-0.62,0.07	0.116			
**LOS_{HOSP}**	-0.36	-0.58,-0.14	**0.001**	-0.31	-0.54,-0.07	**0.010**
**Steroid administration**	-6.00	-18.03,6.02	0.327			
**NMB administration**	2.34	-9.95,14.64	0.707			
**RRT in ICU**	4.89	-14.36,24.14	0.618			
**Tracheostomy in ICU**	-12.99	-24.00,-1.98	**0.021**			
**MV duration**	-0.19	-0.64,0.26	0.405			

	**Univariable Analysis**			**Multivariable Analysis**		
**RE**	**Beta**	**95% CI**	**p-value**	**Beta**	**95% CI**	**p-value**
**Age**	-0.09	-0.54,0.36	0.687			
**Female sex**	-4.68	-15.50,6.14	0.395			
**High level of education**	-2.51	-11.58,6.56	0.587			
**Married/Cohabiting**	-4.03	-14.46,6.41	0.448			
**Employment**						
Active worker vs jobless	-2.68	-19.62,14.26	0.756			
Retired vs jobless	2.90	-14.06,19.87	0.736			
Retired vs active worker	5.58	-3.73,14.89	0.239			
**Weight at baseline**	-0.32	-0.57,-0.06	**0.015**	-0.31	-0.56,-0.06	**0.017**
**Weight at FUP**	-0.45	-0.76,-0.13	**0.005**			

**Weight variation**	0.70	-0.09,1.48	0.082			
**APACHE II score**	0.31	-0.89,1.52	0.607			
**ALI score**	-5.37	-15.49,4.75	0.297			
**Cardiovascular disease**	-12.14	-21.06,-3.13	**0.008**	-9.81	-18.93,-0.68	**0.035**
**Pulmonary disease**	-13.04	-25.21,-0.87	**0.036**	-12.51	-24.94,-0.09	**0.048**
**Kidney disease**	-3.75	-24.42,16.91	0.721			
**Liver disease**	-18.50	-44.68,7.67	0.165			
**Diabetes**	-3.74	-17.66,10.18	0.598			
**Length of hospital stay before ICU admission**	0.60	-0.56,1.76	0.308			
**LOS_{ICU}**	0.13	-0.20,0.46	0.434			
**LOS_{HOSP}**	-0.01	-0.21,0.21	0.974			
**Steroid administration**	-1.96	-13.43,9.51	0.737			
**NMB administration**	-2.85	-15.42,9.73	0.656			
**RRT in ICU**	8.67	-9.55,26.90	0.350			
**Tracheostomy in ICU**	0.55	-10.06,11.17	0.918			
**MV duration**	0.19	-0.23,0.62	0.372			

	**Univariable Analysis**			**Multivariable Analysis**		
**BP**	**Beta**	**95% CI**	**p-value**	**Beta**	**95% CI**	**p-value**
**Age**	-0.20	-0.53,0.12	0.211			
**Female sex**	-6.78	-14.53,0.98	0.087			
**High level of education**	-2.12	-8.86,4.61	0.535			
**Married/Cohabiting**	0.53	-7.18,8.24	0.892			
**Employment**						
Active worker vs jobless	3.23	-9.21,15.67	0.610	3.19	-8.94,15.31	0.606
Retired vs jobless	10.13	-2.33,22.59	0.111	10.82	-1.32,22.95	0.080
Retired vs active worker	6.89	0.05,13.74	**0.048**	7.63	1.10,14.16	**0.022**
**Weight at baseline**	-0.08	-0.26,0.11	0.418			

**Weight at FUP**	-0.12	-0.35,0.11	0.302			
**Weight variation**	0.20	-0.38,0.78	0.494			
**APACHE II score**	-0.34	-1.23,0.55	0.449			
**ALI score**	-5.51	-13.11,2.08	0.154			
**Cardiovascular disease**	-5.05	-11.51,1.41	0.125			
**Pulmonary disease**	-14.49	-23.16,-5.82	**0.001**	-15.76	-24.43,-7.10	**<0.001**
**Kidney disease**	-1.64	-16.50,13.23	0.829			
**Liver disease**	-7.33	-26.19,11.53	0.445			
**Diabetes**	-2.26	-12.25,7.74	0.657			
**Length of hospital stay before ICU admission**	-0.60	-1.43,0.23	0.158			
**LOS_{ICU}**	0.04	-0.20,0.27	0.758			
**LOS_{HOSP}**	-0.09	-0.25,0.06	0.219			
**Steroid administration**	-5.13	-13.68,3.06	0.213			
**NMB administration**	0.23	-8.82,9.28	0.960			
**RRT in ICU**	1.73	-11.67,15.14	0.799			
**Tracheostomy in ICU**	0.72	-6.91,8.36	0.852			
**MV duration**	0.07	-0.24,0.38	0.648			

	**Univariable Analysis**			**Multivariable Analysis**		
**GH**	**Beta**	**95% CI**	**p-value**			
**Age**	-0.03	-0.28,0.23	0.830			
**Female sex**	-7.24	-13.35,-1,13	**0.020**	-5.47	-11.84,0.91	0.093
**High level of education**	-3.47	-8.62,1.69	0.187			
**Married/Cohabiting**	2.86	-3.07,8.79	0.344			
**Employment**						
Active worker vs jobless	12.83	3.30,22.36	**0.008**	4.06	-6.18,14.29	0.436
Retired vs jobless	14.15	4.61,23.69	**0.004**	8.46	-1.72,18.64	0.103

Retired vs active worker	1.31	-3.92,6.55	0.622			
**Weight at baseline**	-0.02	-0.17,0.12	0.756			
**Weight at FUP**	-0.02	-0.18,0.15	0.860			
**Weight variation**	0.15	-0.27,0.57	0.495			
**APACHE II score**	-0.19	-0.87,0.49	0.582			
**ALI score**	-3.41	-9.15,2.34	0.244			
**Cardiovascular disease**	-6.05	-11.13,-0.96	**0.020**	-6.04	-11.21,-0.88	**0.022**
**Pulmonary disease**	-9.25	-16.14,-2.35	**0.009**	-8.47	-15.39,-1.55	**0.017**
**Kidney disease**	-5.56	-17.30,6.18	0.352			
**Liver disease**	-15.11	-29.95,-0.27	**0.046**	-12.36	-27.06,2.34	0.099
**Diabetes**	-4.33	-12.19,3.52	0.279			
**Length of hospital stay before ICU admission**	-0.24	-0.90,0.41	0.465			
**LOS_{ICU}**	0.10	-0.09,0.28	0.320			
**LOS_{HOSP}**	0.01	-0.12,0.12	0.968			
**Steroid administration**	-0.76	-7.25,5.73	0.818			
**NMB administration**	2.00	-4.68,8.68	0.556			
**RRT in ICU**	-2.17	-12.52,8.17	0.680			
**Tracheostomy in ICU**	-0.92	-6.95,5.12	0.765			
**MV duration**	0.19	-0.05,0.44	0.121			

	**Univariable Analysis**			**Multivariable Analysis**		
**VT**	**Beta**	**95% CI**	**p-value**	**Beta**	**95% CI**	**p-value**
**Age**	-0.23	-0.46,-0.01	**0.042**	-0.21	-0.47,0.05	0.111
**Female sex**	-8.83	-14.27,-3.39	**0.002**	-8.10	-13.77,-2.42	**0.005**
**High level of education**	-0.18	-4.83,4.47	0.940			
**Married/Cohabiting**	-2.19	-7.56,3.17	0.422			
**Employment**						

Active worker vs jobless	11.79	3.15,20.43	**0.008**	4.47	-4.68,13.62	0.337
Retired vs jobless	11.30	2.65,19.95	**0.011**	7.28	-1.70,16.26	0.112
Retired vs active worker	-0.49	-5.24,4.26	0.838	2.81	-2.38,8.00	0.287
**Weight at baseline**	-0.03	-0.16,0.10	0.659			
**Weight at FUP**	-0.07	-0.23,0.09	0.408			
**Weight variation**	0.18	-0.22,0.59	0.379			
**APACHE II score**	-0.18	-0.77,0.42	0.559			
**ALI score**	1.07	-4.04,6.18	0.679			
**Cardiovascular disease**	-6.57	-11.12,-2.03	**0.005**	-6.09	-10.79,-1.39	**0.011**
**Pulmonary disease**	-5.75	-11.97,0.46	0.070			
**Kidney disease**	-4.99	-15.52,5.54	0.352			
**Liver disease**	-12.92	-26.24,0.39	0.057			
**Diabetes**	-4.30	-11.23,2.62	0.222			
**Length of hospital stay before ICU admission**	-0.18	-0.77,0.41	0.556			
**LOS_{ICU}**	0.05	-0.12,0.21	0.587			
**LOS_{HOSP}**	-0.07	-0.18,0.04	0.210			
**Steroid administration**	-4.79	-10.58,1.00	0.105			
**NMB administration**	4.08	-2.49,10.65	0.223			
**RRT in ICU**	2.37	-6.88,11.62	0.615			
**Tracheostomy in ICU**	-0.70	-6.11,4.72	0.800			
**MV duration**	0.01	-0.21,0.23	0.922			

	**Univariable Analysis**			**Multivariable Analysis**		
**SF**	**Beta**	**95% CI**	**p-value**	**Beta**	**95% CI**	**p-value**
**Age**	-0.22	-0.52,0.08	0.157			
**Female sex**	-5.56	-12.90,1.78	0.137			

**High level of education**	-4.95	-11.18,1.28	0.119			
**Married/Cohabiting**	-1.18	-8.34,5.98	0.746			
**Employment**						
Active worker vs jobless						
Retired vs jobless	0.57	-11.07,12.22	0.923			
Retired vs active worker	1.25	-10.41,12.91	0.833			
	0.68	-5.72,7.08	0.835			
**Weight at baseline**	-0.09	-0.27,0.08	0.293			
**Weight at FUP**	-0.12	-0.32,0.07	0.223			
**Weight variation**	0.40	-0.08,0.88	0.103			
**APACHE II score**	-0.71	-1.54,0.12	0.092			
**ALI score**	-6.46	-13.49,0.56	0.071			
**Cardiovascular disease**	-6.82	-12.91,-0.73	**0.028**	-6.58	-12.69,-0.48	**0.035**
**Pulmonary disease**	-7.38	-15.67,0.91	0.081			
**Kidney disease**	-9.87	-23.88,4.15	0.167			
**Liver disease**	-8.24	-26.07,9.58	0.364			
**Diabetes**	0.39	-9.10,9.89	0.935			
**Length of hospital stay before ICU admission**	-0.50	-1.29,0.28	0.207			
**LOS_{ICU}**	-0.13	-0.36,0.09	0.238			
**LOS_{HOSP}**	-0.15	-0.30,-0.01	**0.032**	-0.15	-0.29,-0.01	**0.042**
**Steroid administration**	-1.68	-9.59,6.24	0.677			
**NMB administration**	-0.63	-8.60,7.33	0.875			
**RRT in ICU**	1.41	-11.30,14.12	0.827			
**Tracheostomy in ICU**	-5.72	-12.91,1.47	0.119			
**MV duration**	-0.12	-0.42,0.17	0.417			

	Univariable Analysis			Multivariable Analysis		
**MH**	**Beta**	**95% CI**	**p-value**	**Beta**	**95% CI**	**p-value**
**Age**	-0.23	-0.45,-0.01	**0.039**	-0.19	-0.55,0.17	0.297
**Female sex**	-8.41	-13.73,-3.10	**0.002**	-6.22	-13.85,1.41	0.109
**High level of education**	-0.23	-4.83,4.38	0.923			
**Married/Cohabiting**	-1.46	-6.75,3.83	0.588			
**Employment**						
Active worker vs jobless	12.87	4.37,21.37	**0.003**	11.55	-1.21,24.31	0.076
Retired vs jobless	11.48	2.96,20.0	**0.008**	12.30	-0.90,25.50	0.068
Retired vs active worker	-1.39	-6.07,3.29	0.560	0.74	-6.72,8.21	0.844
**Weight at baseline**	-0.02	-0.15,0.11	0.755			
**Weight at FUP**	-0.07	-0.23,0.08	0.352			
**Weight variation**	0.09	-0.30,0.49	0.653			
**APACHE II score**	-0.12	-0.73,0.48	0.695			
**ALI score**	-0.07	-5.26,5.12	0.978			
**Cardiovascular disease**	-2.34	-6.82,2.14	0.306			
**Pulmonary disease**	-9.33	-15.34,-3.31	**0.002**	-8.51	-17.42,0.41	0.061
**Kidney disease**	-7.90	-18.15,2.34	0.130			
**Liver disease**	-2.97	-16.03,10.08	0.655			
**Diabetes**	2.21	-4.64,9.07	0.526			
**Length of hospital stay before ICU admission**	0.03	-0.55,0.60	0.928			
**LOS_{ICU}**	0.11	-0.06,0.27	0.201			
**LOS_{HOSP}**	0.00	-0.10,0.11	0.975			
**Steroid administration**	-6.04	-11.78,-0.29	**0.039**	-0.97	-9.79,7.85	0.829
**NMB administration**	6.78	0.29,13.28	**0.041**	4.48	-2.20,11.15	0.187

**RRT in ICU**	4.88	-4.50,14.26	0.307			
**Tracheostomy in ICU**	1.15	-4.15,6.46	0.669			
**MV duration**	0.08	-0.14,0.30	0.486			

	**Univariable Analysis**			**Multivariable Analysis**		
**Health change**	**Beta**	**95% CI**	**p-value**	**Beta**	**95% CI**	**p-value**
**Age**	-0.25	-0.53,0.03	0.083			
**Female sex**	-3.71	-10.52,3.10	0.285			
**High level of education**	3.49	-3.14,10.13	0.301			
**Married/Cohabiting**	-2.46	-10.09,5.17	0.527			
**Employment**						
Active worker vs jobless	14.20	1.93,26.48	**0.023**	9.58	-7.25,26.41	0.263
Retired vs jobless	6.16	-6.13,18.45	0.325	5.97	-10.54,22.48	0.476
Retired vs active worker	-8.04	-14.79,-1.29	**0.020**	-3.61	-11.63,4.41	0.376
**Weight at baseline**	-0.04	-0.20,0.12	0.590			
**Weight at FUP**	-0.11	-0.31,0.10	0.311			
**Weight variation**	0.30	-0.21,0.81	0.244			
**APACHE II score**	-0.28	-0.99,0.44	0.449			
**ALI score**	-3.09	-9.98,2.17	0.207			
**Cardiovascular disease**	-6.82	-12.45,-1.18	**0.018**	-7.19	-15.47,1.10	0.089
**Pulmonary disease**	-3.49	-11.20,4.21	0.374			
**Kidney disease**	-5.17	-18.18,7.84	0.435			
**Liver disease**	-10.33	-26.84,6.17	0.219			
**Diabetes**	-8.92	-17.68,-0.16	**0.046**	-8.93	-19,24,1.39	0.089
**Length of hospital stay before ICU admission**	0.00	-0.73,0.71	0.993			
**LOS_{ICU}**	0.05	-0.16,0.25	0.652			

**LOS_{HOSP}**	-0.11	-0.24,0.02	0.114			
**Steroid administration**	-0.38	-0.87,0.12	0.136			
**NMB administration**	8.83	0.75,16.91	**0.032**	8.67	0.39,16.94	**0.040**
**RRT in ICU**	-8.91	-20.01,2.19	0.115			
**Tracheostomy in ICU**	3.46	-3.21,10.14	0.309			
**MV duration**	0.11	-0.16,0.38	0.427			

	**Univariable**			**Multivariable**		
**Physical Component Summary**	**Beta**	**95% CI**	**p-value**	**Beta**	**95% CI**	**p-value**
**Age**	-0.12	-0.24,-0.04	**0.043**	-0.11	-0.25,0.02	0.093
**Female sex**	-2.81	-5.66,0.04	0.053			
**High level of education**	0.71	-1.64,3.07	0.551			
**Married/Cohabiting**	-0.41	-3.12,2.30	0.768			
**Employment**						
Active worker vs jobless	6.27	1.92,10.62	**0.005**	3.82	-0.74,8.38	0.100
Retired vs jobless	6.87	2.51,11.23	**0.002**	6.42	1.93,10.92	**0.005**
Retired vs active worker	0.60	-1.79,2.98	0.623	2.60	-0.05,5.26	0.054
**Weight at baseline**	0.01	-0.07,0.07	0.971			
**Weight at FUP**	-0.01	-0.08,0.08	0.997			
**Weight variation**	0.05	-0.15,0.26	0.599			
**APACHE II score**	-0.19	-0.50,0.13	0.243			
**ALI score**	-0.85	-3.54,1.84	0.535			
**Cardiovascular disease**	-2.85	-5.19,-0.50	**0.018**	-2.23	-4.64,0.17	0.069
**Pulmonary disease**	-3.84	-7.02,-0.67	**0.018**	-2.87	-6.03,0.29	0.075
**Kidney disease**	-1.16	-6.50,4.19	0.671			
**Liver disease**	-9.32	-16.40,-2.25	**0.010**	-8.58	-15.43,-1.73	**0.014**

**Diabetes**	-2.99	-6.71,0.73	0.114			
**Length of hospital stay before ICU admission**	-0.12	-0.42,0.18	0.438			
**LOS_{ICU}**	-0.04	-0.13,0.04	0.325			
**LOS_{HOSP}**	-0.08	-0.14,-0.03	**0.003**	-0.07	-0.13,-0.02	**0.007**
**Steroid administration**	-0.85	-3.82,2.12	0.572			
**NMB administration**	1.50	-1.78,4.79	0.368			
**RRT in ICU**	-1.04	-5.88,3.80	0.673			
**Tracheostomy in ICU**	-1.98	-4.81,0.85	0.170			
**MV duration**	-0.01	-0.12,0.11	0.915			

	**Univariable**		
**Mental Component Summary**	**Beta**	**95% CI**	**p-value**
**Age**	0.04	-0.02,0.09	0.224
**Female sex**	-0.01	-1.41,1.40	0.993
**High level of education**	-0.74	-1.91,0.44	0.220
**Married/Cohabiting**	0.54	-0.83,1.91	0.442
**Employment**			
Active worker vs jobless	-2.25	-4.46,-0.03	**0.047**
Retired vs jobless	-2.43	-4.64,-0.21	**0.032**
Retired vs active worker	-0.018	-1.39,1.03	0.771
**Weight at baseline**	0.01	-0.02,0.05	0.425
**Weight at FUP**	0.02	-0.03,0.06	0.415
**Weight variation**	-0.03	-0.14,0.08	0.635
**APACHE II score**	0.03	-0.13,0.19	0.688

**ALI score**	0.38	-0.99,1.75	0.587
**Cardiovascular disease**	0.54	-0.62,1.69	0.362
**Pulmonary disease**	1.33	-0.23,2.89	0.094
**Kidney disease**	-0.06	-2.68,2.55	0.962
**Liver disease**	1.58	-1.91,5.07	0.374
**Diabetes**	0.64	-1.19,2.47	0.491
**Length of hospital stay before ICU admission**	-0.12	-0.27,0.03	0.105
**LOS_{ICU}**	-0.01	-0.05,0.03	0.667
**LOS_{HOSP}**	0.01	-0.01,0.04	0.317
**Steroid administration**	0.13	-1.35,1.61	0.862
**NMB administration**	-0.84	-2.62,0.95	0.356
**RRT in ICU**	0.42	-1.99,2.83	0.734
**Tracheostomy in ICU**	0.30	-1.09,1.69	0.670
**MV duration**	-0.03	-0.09,0.02	0.234

Data are expressed as linear regression coefficient (β) and confidence intervals (95% CI).

Negative beta values means lower SF-36 score, so a reduced HRQoL and vice versa.

Legend: FUP=follow-up, APACHE II score=Acute Physiologic Assessment and Chronic Health Evaluation II score, ALI score=Acute Lung Injury score, ICU=Intensive Care Unit, LOS_{ICU}=length of ICU stay, LOS_{HOSP}=length of hospital stay, NMB=neuromuscular blockers, RRT=renal replacement therapy, MV=mechanical ventilation, PCS=physical component summery, MCS=mental component summary.

Supplementary Material S3.

Univariable and multivariable analysis to find possible correlations between IES-R items and baseline, medical and ICU data.

	**Univariable analysis**			**Multivariable analysis**		
**PTSD PRESENCE**	**OR**	**95% CI**	**p-value**	**OR**	**95% CI**	**p-value**
**Age**	0.99	0.97,1.02	0.642			

**Female sex**	1.55	0.87,2.79	0.135			
**High level of education**	0.99	0.62,1.57	0.966			
**Married/Cohabiting**	0.72	0.43,1.21	0.219			
**Employment**						
Active worker vs jobless	0.70	0.31,1.59	0.402			
Retired vs jobless	0.49	0.21,1.12	0.092			
Retired vs active worker	0.70	0.43,1.13	0.141			
**Weight at baseline**	1.01	0.99,1.03	0.059			
**Weight at FUP**	1.02	1.00,1.04	**0.047**	1.02	1.00,1.04	**0.030**
**Weight variation**	0.96	0.92,1.01	0.083			
**APACHE II score**	1.02	0.94,1.09	0.653			
**LIS**	1.46	0.80,2.65	0.216			
**Cardiovascular disease**	2.14	1.28,3.55	**0.003**	1.58	0.86,2.92	0.142
**Pulmonary disease**	1.76	0.92,3.38	0.088			
**Kidney disease**	3.70	1.27,10.83	**0.017**	2.74	0.84,8.88	0.093
**Liver disease**	3.40	0.84,13.79	0.086			
**Diabetes**	0.82	0.37,1.81	0.624			
**Length of hospital stay before ICU admission**	1.07	0.99,1.14	0.051			
**LOS_{ICU}**	0.98	0.96,1.00	0.103			
**LOS_{HOSP}**	1.00	0.99,1.01	0.728			
**Steroid administration**	1.19	0.61,2.31	0.603			
**NMB administration**	1.00	0.50,1.98	0.995			
**RRT in ICU**	1.15	0.41,3.25	0.786			
**Tracheostomy in ICU**	0.70	0.38,1.28	0.248			
**MV duration**	0.98	0.96,1.01	0.261			

	Univariable analysis			Multivariable analysis		
**AVOIDANCE**	**Beta**	**95% CI**	**p-value**	**Beta**	**95% CI**	**p-value**
**Age**	-0.01	-0.02,0.00	0.145			
**Female sex**	0.39	0.16,0.62	**0.001**	0.31	0.06,0.56	**0.015**
**High level of education**	-0.07	-0.27,0.12	0.471			
**Married/Cohabiting**	0.02	-0.20,0.25	0.842			
**Employment**						
Active worker vs jobless	-0.05	-0.41,0.32	0.802			
Retired vs jobless	-0.21	-0.58,0.15	0.254			
Retired vs active worker	-0.17	-0.37,0.04	0.107			
**Weight at baseline**	0.01	0.00,0.01	0.070			
**Weight at FUP**	0.00	0.00,0.01	0.188			
**Weight variation**	-0.02	-0.04,-0.01	**0.010**	-0.02	-0.03,0.00	**0.025**
**APACHE II score**	0.01	-0.02,0.03	0.727			
**LIS**	0.11	-0.11,0.32	0.326			
**Cardiovascular disease**	0.23	0.04,0.43	**0.017**	-0.01	-0.23,0.21	0.904
**Pulmonary disease**	0.40	0.14,0.66	**0.003**	0.30	0.00,0.59	**0.047**
**Kidney disease**	0.82	0.39,1.25	**<0.001**	0.50	0.04,0.96	**0.031**
**Liver disease**	0.45	-0.11,1.00	0.115			
**Diabetes**	-0.11	-0.41,0.19	0.464			
**Length of hospital stay before ICU admission**	0.03	0.01,0.06	**0.005**			
**LOS_{ICU}**	0.00	-0.01,0.01	0.900			
**LOS_{HOSP}**	0.01	0.00,0.01	**0.029**	0.01	-0.01,0.01	0.057
**Steroid administration**	0.05	-0.20,0.29	0.709			
**NMB administration**	-0.03	-0.28,0.23	0.832			
**RRT in ICU**	0.23	-0.15,0.61	0.237			
**Tracheostomy in ICU**	-0.01	-0.24,0.22	0.954			

**MV duration**	0.00	-0.01,0.01	0.916			

	**Univariable analysis**			**Multivariable analysis**		
**INTRUSIVITY**	**Beta**	**95% CI**	**p-value**	**Beta**	**95% CI**	**p-value**
**Age**	-0.01	-0.02,0.00	**0.035**	-0.00	-0.02,0.01	0.556
**Female sex**	0.36	0.08,0.63	**0.011**	0.27	-0.05,0.58	0.094
**High level of education**	-0.03	-0.27,0.21	0.830			
**Married/Cohabiting**	-0.01	-0.29,0.26	0.911			
**Employment**						
Active worker vs jobless	-0.13	-0.57,0.32	0.576	-0.12	-0.65,0.41	0.644
Retired vs jobless	-0.44	-0.89,0.00	0.051	-0.53	-1.06,0.08	0.053
Retired vs active worker	-0.31	-0.56,-0.07	**0.012**	-0.40	-0.71,-0.10	**0.010**
**Weight at baseline**	0.01	0.00,0.01	0.140			
**Weight at FUP**	0.01	0.00,0.01	0.132			
**Weight variation**	-0.03	-0.05,-0.01	**<0.001**	-0.04	-0.05,-0.02	**<0.001**
**APACHE II score**	-0.01	-0.04,0.02	0.574			
**LIS**	0.27	0.02,0.53	0.051			
**Cardiovascular disease**	0.14	-0.09,0.37	0.244			
**Pulmonary disease**	0.38	0.07,0.70	**0.017**	0.24	-0.11,0.58	0.178
**Kidney disease**	0.83	0.31,1.35	**0.002**	0.55	0.02,1.08	**0.042**
**Liver disease**	0.41	-0.26,1.07	0.232			
**Diabetes**	-0.20	-0.56,0.15	0.268			
**Length of hospital stay before ICU admission**	0.02	-0.01,0.05	0.118			
**LOS_{ICU}**	0.00	-0.01,0.01	0.843			
**LOS_{HOSP}**	0.01	0.00,0.01	0.065			
**Steroid administration**	0.05	-0.25,0.35	0.756			

**NMB administration**	0.05	-0.26,0.36	0.759			
**RRT in ICU**	0.17	-0.30,0.64	0.486			
**Tracheostomy in ICU**	0.09	-0.18,0.37	0.519			
**MV duration**	0.00	-0.01,0.01	0.669			

	**Univariable analysis**			**Multivariable analysis**		
**HYPERAROUSAL**	**Beta**	**95% CI**	**p-value**	**Beta**	**95% CI**	**p-value**
**Age**	-0.01	-0.02,0.00	0.192			
**Female sex**	0.40	0.14,0.66	**0.003**	0.26	-0.06,0.58	0.110
**High level of education**	-0.08	-0.31,0.15	0.500			
**Married/Cohabiting**	-0.01	-0.28,0.25	0.915			
**Employment**						
Active worker vs jobless	-0.26	-0.68,0.16	0.220	-0.18	-0.72,0.36	0.510
Retired vs jobless	-0.52	-0.94,-0.10	**0.016**	-0.46	-0.99,0.08	0.093
Retired vs active worker	-0.25	-0.49,-0.02	**0.033**	-0.28	-0.54,-0.02	**0.036**
**Weight at baseline**	0.01	0.00,0.01	**0.006**			
**Weight at FUP**	0.01	0.00,0.02	**0.014**			
**Weight variation**	-0.03	-0.05,-0.01	**0.003**	-0.03	-0.05,-0.01	**0.001**
**APACHE II score**	-0.01	-0.03,0.03	0.921			
**LIS**	0.08	-0.16,0.32	0.497			
**Cardiovascular disease**	0.16	-0.05,0.38	0.140			
**Pulmonary disease**	0.18	-0.12,0.48	0.233			
**Kidney disease**	0.76	0.27,1.25	**0.003**	0.42	-0.11,0.95	0.123
**Liver disease**	0.48	-0.15,1.11	0.134			
**Diabetes**	-0.01	-0.35,0.32	0.927			
**Length of hospital stay before ICU admission**	0.02	-0.01,0.04	0.230			

**LOS_{ICU}**	0.00	-0.01,0.01	0.547			
**LOS_{HOSP}**	0.00	0.00,0.01	0.513			
**Steroid administration**	0.02	-0.26,0.30	0.913			
**NMB administration**	-0.07	-0.38,0.24	0.662			
**RRT in ICU**	0.03	-0.41,0.47	0.892			
**Tracheostomy in ICU**	0.03	-0.23,0.29	0.821			
**MV duration**	0.00	-0.01,0.01	0.930			

	**Univariable analysis**			**Multivariable analysis**		
**GLOBAL MEAN**	**Beta**	**95% CI**	**p-value**	**Beta**	**95% CI**	**p-value**
**Age**	-0.01	-0.02,0.00	0.142			
**Female sex**	0.45	0.15,0.74	**0.003**	0.27	-0.11,0.65	0.167
**High level of education**	-0.03	-0.28,0.21	0.789			
**Married/Cohabiting**	-0.01	-0.29,0.28	0.959			
**Employment**						
Active worker vs jobless	-0.23	-0.70,0.23	0.320	-0.09	-0.72,0.54	0.772
Retired vs jobless	-0.48	-0.94,-0.01	**0.043**	-0.59	-1.22,0.04	0.067
Retired vs active worker	-0.24	-0.50,0.01	0.061	-0.49	-0.81,-0.18	**0.002**
**Weight at baseline**	0.01	0.00,0.01	0.115			
**Weight at FUP**	0.00	0.00,0.01	0.305			
**Weight variation**	-0.02	-0.05,0.00	**0.019**	-0.02	-0.05,0.00	**0.049**
**APACHE II score**						
**LIS**						
**Cardiovascular disease**	0.14	-0.11,0.38	0.284			
**Pulmonary disease**	0.25	-0.09,0.58	0.148			
**Kidney disease**	0.89	0.33,1.45	**0.002**	0.65	-0.08,1.37	0.082
**Liver disease**	0.48	-0.24,1.20	0.188			

**Diabetes**	-0.07	-0.45,0.31	0.718			
**Length of hospital stay before ICU admission**	0.03	0.00,0.06	**0.045**	0.03	0.00,0.07	0.073
**LOS_{ICU}**	0.00	-0.01,0.00	0.279			
**LOS_{HOSP}**	0.00	0.00,0.01	0.736			
**Steroid administration**	0.33	0.01,0.65	**0.043**	-0.05	-0.51,0.41	0.834
**NMB administration**	-0.02	-0.28,0.24	0.859			
**RRT in ICU**	0.08	-0.44,0.59	0.766			
**Tracheostomy in ICU**	-0.07	-0.37,0.22	0.613			
**MV duration**	0.00	-0.01,0.01	0.614			

Data are expressed as linear regression coefficient (β) and confidence intervals (95% CI).

Legend: FUP=follow-up, APACHE II score=Acute Physiologic Assessment and Chronic Health Evaluation II score, LIS=Lung Injury Score, ICU=intensive care unit, LOS_{ICU}=length of ICU stay, LOS_{HOSP}=length of hospital stay, NMB=neuromuscular blockers, RRT=renal replacement therapy, MV=mechanical ventilation.
